# The Role of Vitamin D Deficiency and Vitamin D Receptor Genotypes on the Degree of Collateralization in Patients with Suspected Coronary Artery Disease

**DOI:** 10.1155/2014/304250

**Published:** 2014-03-06

**Authors:** Arash Hossein-Nezhad, Seyede Mahdieh Eshaghi, Zhila Maghbooli, Khadijeh Mirzaei, Mahmood Shirzad, Bryon Curletto, Tai C. Chen

**Affiliations:** ^1^Department of Medicine, Section of Endocrinology, Nutrition, and Diabetes, Vitamin D, Skin, and Bone Research Laboratory, Boston University Medical Center, Boston, MA 02118, USA; ^2^Tehran University of Medical Sciences, Tehran 1417653911, Iran; ^3^Department of Community Nutrition, School of Nutritional Sciences and Dietetics, Tehran University of Medical Sciences, Tehran 1417653911, Iran; ^4^Tehran Heart Center, Tehran University of Medical Sciences, Tehran 1417653911, Iran

## Abstract

We determined the association of vitamin D deficiency and the FokI polymorphism of the vitamin D receptor (VDR) gene in 760 patients who underwent angiography due to suspected coronary artery disease (CAD). Angiography and the Rentrop scoring system were used to classify the severity of CAD in each patient and to grade the extent of collateral development, respectively. Polymerase chain reaction-restriction fragment length polymorphism (PCR-RFLP) was used to determine the FokI VDR gene polymorphism. The prevalence of severe vitamin D deficiency (serum 25(OH)D < 10 ng/mL) was significantly higher in patients with at least one stenotic coronary artery compared to those without any stenotic coronary arteries. Severe vitamin D deficiency was not independently associated with collateralization, but it was significantly associated with the VDR genotypes. In turn, VDR genotype was independently associated with the degree of collateralization; the Rentrop scores were the highest in FF, intermediate in Ff, and the lowest in the ff genotype. The results show that FokI polymorphism is independently associated with collateralization. Additionally, vitamin D deficiency is more prevalent in patients with CAD that may result from FokI polymorphism. Therefore, maintaining a normal vitamin D status should be a high priority for patients with CAD.

## 1. Introduction

The active form of vitamin D, 1**α**,25-dihydroxyvitamin D [1**α**,25(OH)_2_D], plays an important role in the endocrine system by exerting its influence on various metabolic pathways. It has been documented that vitamin D deficiency is associated with a constellation of chronic disorders such as autoimmune diseases, certain cancers, and osteoporosis [1]. Recent studies have demonstrated that vitamin D deficiency is independently associated with the prevalence of cardiovascular disease [2]. Vitamin D deficiency has also been associated with an increased risk of incident hypertension [3], diabetes [3], and hyperlipidemia [4], which are the known risk factors for the development of cardiovascular disease [4–8]. Numerous experimental and epidemiological studies have demonstrated that the levels of the circulating form of vitamin D, 25-hydroxyvitamin D_3_ [25(OH)D_3_], are inversely associated with increased mortality from cardiovascular and heart diseases [9–11]. Vitamin D supplementation has been shown to have some protective value in the primary prevention of coronary heart disease [12]. Most importantly, a growing body of evidence indicates that maintaining the serum 25(OH)D_3_ levels within the normal range may be beneficial for general health [10], suggesting that measuring serum levels of 25(OH)D_3_ may be clinically valuable to predict patients' susceptibility to coronary artery disease [13, 14]. There are compelling lines of evidence which confirmed that 25(OH)D_3_ levels play a major role in the health of a wide variety of organ systems [15]. An inverse association between vitamin D deficiency and the prevalence of cardiometabolic risk factors, such as type 2 diabetes, hypertension, dyslipidaemia, and the metabolic syndrome has been reported [14]. Moreover, the concentration of 25(OH)D_3_ is considered as a clinical valuable marker to predict the risk of CAD. Vitamin D deficiency has been associated with an increased risk of CAD and cardiovascular death. Endothelial dysfunction plays an important role in pathogenesis of CAD and vitamin D deficiency is postulated to promote endothelial dysfunction. According to previous studies, patients with vitamin D deficiency had also significantly higher prevalence of double- or triple-vessel CAD, diffuse CAD, and higher number of coronary vessels involved as compared to those with higher 25(OH)D_3_ levels [13].

The biological actions of vitamin D are mediated through the nuclear vitamin D receptor (VDR) [16]. VDR forms a heterodimer with the retinoid X receptor (RXR) while 1**α**,25(OH)_2_D acts as a ligand forming a 1**α**,25(OH)2D/VDR complex. This ligand-bound VDR complex then binds to a sequence or sequences, called VDR response elements (VDREs), in the promoter region of target genes and acts as a transcription factor to promote gene expression. The precise mechanism of how the ligand/VDR complex may help protect individuals against cardiovascular diseases is not clear. It has been documented that vitamin D has functional roles in cellular proliferation, including different stages of the cell cycle, differentiation, and apoptosis [17, 18] that could have impact on normal angiogenesis and endothelial cell differentiation [19]. VDR is expressed in almost all tissues and in a wide variety of cell types, including cardiomyocytes, vascular smooth muscle cells, and endothelial cells [20, 21]. In addition, macrophages, monocytes, endothelial cells, and vascular smooth muscle cells have the capability to activate vitamin D locally in an autocrine fashion [22]. The presence of VDR in vascular smooth muscle cells and endothelial cells coupled with the ability of vascular tissues to activate vitamin D locally suggests a role vitamin D may play in normal vascular physiology and its role in the prevention of ischemic heart disease.

Coronary collateral arteries are believed to improve the prognosis of patients with ischemic heart diseases like CAD by preserving perfusion and function of the myocardium [23]. In this study, our aims are to determine whether vitamin D deficiency is associated with collateralization and whether the* FokI* polymorphism of the VDR gene could affect collateralization in CAD patients. Understanding the underlying molecular genetics involved in the collateral development may be clinically valuable to formulate prevention strategies for patients who are at an increased risk for CAD.

Likewise, in order to better understand the role of the ligand-bound VDR in the degree of collateralization the role of specific elements of the VDR gene in modifying VDR function should be considered. Previously, we have provided evidence indicating that different VDR genotypes may be associated with different functions of vitamin D [23, 24]. Among the multiple polymorphisms of the VDR gene,* FokI* polymorphism (rs10735810) is the only one without any link to other VDR polymorphisms and is the sole polymorphism that leads to two different VDR protein products [25, 26]. In the presence of a* FokI* polymorphism, an alternative in-frame start codon, located 10 nucleotides downstream, initiates transcription. The* FokI* polymorphism causes transcription to begin at the second ATG start codon in the exon 2 and ultimately results in the translation of a potentially shortened receptor protein. The F allele codes 3 amino acid shorter VDR sequence than that coded by the f allele. Some studies have argued that the VDR coded by the F allele is more efficient in transactivating target genes [27–29]. According to recent clinical and experimental studies, VDR may indeed play a role in cardiovascular diseases and the VDR gene polymorphism could have an important role in transactivation efficiency of target genes [17, 18].

## 2. Materials and Methods

### 2.1. Patient Selection

The current cross-sectional study included 760 individuals who underwent a clinical assessment and a treatment program between October 2007 and March 2011 at cardiology clinics of major hospitals affiliated to Tehran University of Medical Sciences.* Exclusion criteria* were endocrine disorders, inflammatory diseases, history of previous heart attack, previous cardiac surgery, malignancies, and known chronic diseases with the exception of classic risk factors of CAD. The ethics committee of Endocrinology and Metabolism Research Institute approved the study protocol. At the time of recruitment, all participants provided written informed consent to participate in all stages of the study, including DNA genotyping.

### 2.2. Physical Examination

Clinical information was obtained using a standardized health questionnaire, and a general practitioner performed the physical examination. Body mass index (BMI) was calculated as body weight divided by the square of height expressed in kg/m^2^. Blood pressure was measured two times using a standard calibrated mercury sphygmomanometer on the right arm of participants after they remained seated for 15 minutes. The mean of two blood pressure measurements was recorded as blood pressure. Left ventricular ejection fraction was measured using echocardiography. Angiography was performed on patients who presented with cardiac chest pain and a positive exercise test.

### 2.3. Coronary Angiography and CAD Classification

A cardiology specialist performed the angiogram on each of the patients for whom angiography was indicated. Following angiography, patients were grouped to four classes based on luminal stenosis and the number of coronary arteries involved. In class 0, luminal stenosis was less than 50% and considered normal. In all other classes, where luminal stenosis was more than 50%, the participants were considered CAD patients: In class 1, luminal stenosis was more than 50% in one artery; in class 2, luminal stenosis was more than 50% in two arteries; in class 3, luminal stenosis was more than 50% in three arteries. According to this classification 84.5% of participants were considered CAD patients (classes 1–3).

### 2.4. Coronary Angiography and Collateral Grading/Group

The Rentrop scoring system [30] was used to grade the extent of the degree of collateralization in study subjects: in grade 0, no filling was visible in any collateral channels; in grade 1, collateral filling was visible in side branches of the vessel without dye reaching the epicardial segment of that vessel; in grade 2, partial collateral filling was visible in the epicardial segment of the vessel; in grade 3, complete collateral filling was visible in the vessel. When multiple coronary arteries were involved, the highest Rentrop score was considered as the patient's extent of the degree of collateralization. Based on Rentrop scores, patients were categorized into either a poor-collateralization group (grades 0-1) or a well-collateralization group (grades 2-3).

### 2.5. Blood Sampling

Patients fasted for 12 hours before 12 mL of peripheral venous blood was collected from the patients. Blood samples were added to two aliquots tubes (each 4 mL) for serum preparation and the other 4 mL was added to EDTA tube for DNA isolation. All separated sera and EDTA tubes were stored at −80°C.

### 2.6. Biochemical and Genetic Analyses

Various biochemical analyses were performed in serum obtained from peripheral venous blood after centrifugation at 2,000 ×g for 20 minutes in a refrigerated centrifuge. Serum 25(OH)D was measured by radioimmunoassay (RIA) using a Biosource kit (Biosource Europe SA, Belgium); intra- and interassay coefficients of variation (CV) were 5.2% and 7.5%, respectively. Fasting blood sugar (FBS), triglyceride (TG), total cholesterol (TC), high-density lipoprotein (HDL), and low-density lipoprotein (LDL) levels were measured using a Randox laboratories kit (Hitachi 902). Fasting blood sugar was measured by the GOD/PAP method. Triglyceride was measured by the GPO-PAP method. Total cholesterol was measured by Enzymatic Endpoint. Direct high- and low-density lipoprotein was measured by enzymatic clearance assay. Cardiovascular risk was determined in accordance with the World Health Organization's criteria for cardiovascular risk factors [20]: TC > 200 mg/dL, LDL > 160 mg/dL, TG > 150 mg/dL, FBS > 110, HDL < 35 mg/dL, and hypertensive BP ≥ 140/90 mmHg or patients with their blood pressure controlled by antihypertensive medication.

Genomic DNA extraction was performed using a FlexiGen DNA kit (QIAGEN kit), according to the manufacturer's protocol. The* FokI* polymorphism in exon 2 of the VDR gene was detected by Polymerase chain reaction-restriction fragment length polymorphism (PCR-RFLP). PCR-RFLP was confirmed by repeated analysis, and genotyping was confirmed by sequencing 15% of total PCR products.

### 2.7. Statistical Analysis

Data were analyzed using SPSS software, version 11.5. Student's *t*-test and analysis of variance (ANOVA) compared differences between the means of continuous variables. The chi-square test compared differences between categorical variables. We used univariate analysis from General Linear Model (GLM). In this model, we controlled the effect of confounding variable including age, sex, severity of CAD, and cardiovascular risk factors (higher TG, higher FBS, higher total cholesterol, higher BMI, and lower ejection fraction) on dependent variable through inserting them as covariates. In all tests, the level of significance was set at 0.05 (*P* < 0.05).

## 3. Results

This study included 582 men and 178 women with a total of 760 subjects who underwent angiography. The mean age was 57.97 ± 10.58 years. The clinical and biochemical risk factors of participants are shown in Table 1. The mean serum 25(OH)D level of the participants was 7.2 ± 5.2 ng/mL. Ninety-eight% of the participants had serum 25(OH)D levels less than 20 ng/mL and were considered as vitamin D deficient [31]. Among the vitamin D deficient participants, 70% had serum 25(OH)D levels less than 10 ng/mL and were considered severe vitamin D deficient (62% of women, 72% of men).

*(A) CAD Classification, Cardiovascular Risk Factors, and Vitamin D Deficiency*. Patients with class 3 CAD were older (*P* = 0.005) with higher TG (*P* = 0.01) and lower HDL (*P* = 0.001) compared to other classes. There were no significant associations between CAD classes and hypertension, LDL, TC, or impaired FBS (blood sugar > 110 mg/dL) (Table 1).

Severe vitamin D deficiency (<10 ng/mL) was significantly more frequent in subject with classes 1–3 (CAD patients) than in class 0 (75% versus 56.8%, *P* = 0.007). Class 3 CAD patients had a significantly higher prevalence of severe vitamin D deficiency compared to other classes (*P* = 0.0001) (Table 1).

There were significant associations between severe vitamin D deficiency and hypertension (*P* values = 0.007), impaired FBS (*P* values = 0.007), higher TG (*P* values = 0.002), lower HDL (*P* values = 0.001), and higher TC (*P* values = 0.02).

*(B) CAD Classification, Cardiovascular Risk Factors, and VDR Genotypes*. Demographics regarding genotype are summarized in Table 2. 54.5% of participants were homozygous for the F allele, 7.6% were homozygous for the f allele, and 37.89% were heterozygous. No significant differences in VDR genotypes or allele frequencies were observed between different classes of CAD. Interestingly, patients with ff genotype were significantly younger than those with other genotypes (*P* value = 0.003).

Significant differences in some cardiovascular risk factors were found, including higher TG (*P* = 0.02), higher FBS (*P* = 0.01), higher total cholesterol (*P* = 0.01), and higher BMI (*P* = 0.01) as well as lower ejection fraction (*P* = 0.03) among participants with different genotypes, but there were no significant differences in other risk factors, including smoking (*P* = 0.3), hypertension (*P* = 0.3), lower HDL (*P* = 0.6), or higher LDL (*P* = 0.5).

The prevalence of severe vitamin D deficiency was significantly higher in patients with ff and Ff genotypes compared to patients with FF genotype (*P* = 0.001). 

*(C) CAD Class, Vitamin D Status, VDR Genotype, and the Degree of Collateralization*. The relationship between VDR genotype with CAD classification, vitamin status, and degree of collateralization was summarized in Table 3. There was a significant and linear association between the degree of collateralization and CAD class. The percent of CAD patients in the well-collateralization group increased with CAD class; 50.5% for class 1, 74.8% for class 2, and 92% for class 3.

Our results demonstrate that patients in the poor-collateralization group had a higher prevalence of severe vitamin D deficiency than the well-collateralization group (poor-collateral group; 75.2% versus well-collateral group; 66.5%, *P* < 0.0001).

VDR genotype was significantly associated with collateral development. 80.2% of patients with the FF genotype were in the well-collateralization group compared to only 51.72% of those with the ff genotype who were in the well-collateralization group (*P* = 0.01). Following logistical regression analysis, we found that carriers of the f allele (patients with ff and Ff genotypes) were associated with the poor-collateral group (*P* = 0.004). After adjustment for age, sex, severity of CAD, and cardiovascular risk factors (higher TG, higher FBS, higher total cholesterol, higher BMI, and lower ejection fraction), FF genotype was still significantly associated with the well-collateral group (*P* = 0.02). The relative risk of having poor-collateralization group was 1.5 for patients with ff genotypes (OR: 1.96, 95% CI: 1.14 to 3.37, *P* = 0.019).

## 4. Discussion

The first aim of this study was to determine whether vitamin D deficiency could be associated with the degree of collateralization. Our results showed that vitamin D deficiency was more prevalent in patients with CAD. Ninety-eight% of patients had serum 25(OH)D levels less than 20 ng/mL, and 69.73% of patients had serum vitamin D levels less than 10 ng/mL that is considered as vitamin D deficient and severe vitamin D deficient, respectively. Serum 25(OH)D levels have been shown to be inversely associated with severity of CAD. A growing body of evidence reveals that vitamin D deficiency is more prevalent in patients with ischemic heart disease, suggesting that vitamin D deficient patients may be also more susceptible to cardiovascular diseases [2, 32, 33]. Our findings support the plethora of existing evidence that suggests vitamin D deficiency has a predictive role in the development of CAD [2, 33, 34].

Our data showed that vitamin D deficiency was more common among patients in the poor-collateralization group compared to patients in the well-collateralization group. According to experimental data, vascular tissue expresses VDR and is potentially an important target for vitamin D; therefore, it has been suggested that vitamin D may have a protective role in the vascular system [35]. The precise mechanisms of how vitamin D protects against CAD have not been fully delineated. However, we do know that both vascular cells and immune cells can locally synthesize 1**α**,25(OH)_2_D and that VDR is expressed in both cell types. Taken together, this evidence suggests a biological role for vitamin D in cardiovascular function and angiogenesis [36, 37], such as enhancing collateral development. Thus, the role the vitamin D may play in collateralization is explained by several biologically indirect mechanisms, namely, some of the known cardiovascular risk factors. Our results showed that there was indeed an association between severe vitamin D deficiency and these same three risk factors; hypertension, higher FBS, and higher total cholesterol levels along with two additional risk factors; higher TG and lower HDL.

Recent evidence showed that certain cardiovascular risk factors such as hypertension, hyperglycemia, and hypercholesterolemia could affect the vascular growth processes and impair the normal capacity for vascular growth [38, 39]. Numerous epidemiological studies have also demonstrated that vitamin D deficiency is correlated with the aforementioned risk factors by different mechanisms [40–42].

In one such study regarding the risk factor hypertension, vitamin D has been shown to directly control blood pressure as a negative regulator of rennin-angiotensin system [42]. Regarding hyperglycemia, vitamin D deficiency has been correlated with beta-cell dysfunction and insulin resistance [43]. A meta-analysis of several clinical studies has also shown that lower levels of vitamin D and calcium may impair glucose metabolism and suggested that a combined supplementation of vitamin D with calcium could have a beneficial effect on controlling glucose homeostasis [41]. Prior studies have also demonstrated that low serum levels of vitamin D were associated with obesity, hypertriglyceridemia, and lower HDL levels [44, 45] by some ill-defined mechanisms. Hence, based on the fact that vitamin D deficiency has been associated with certain cardiovascular risk factors, it is likely that vitamin D deficiency could interfere with collateral artery development by altering the complicated process of pathological changes that may be influenced by these very same cardiovascular risk factors.

The second aim of this study was to examine potential relationships between VDR genotype and collateralization in CAD patients. To investigate if the VDR gene plays a role in collateralization, we first investigated relationships between* FokI* polymorphism and severities of CAD, which has a direct effect on collateral development by itself. No significant difference between VDR genotype and severity of CAD was found. Consistent with our result, Pan et al. found no association between* FokI* polymorphism and CAD [46]. Several studies have demonstrated that a wide variety of cells express VDR, suggesting that vitamin D may be involved in regulating different cellular processes, including proliferation, differentiation, and migration [10, 18, 47] that are involved in the progression of CAD [19]. Thus, it is likely the* FokI* polymorphism has a functional role in CAD indirectly via vitamin D deficiency. In addition, our results demonstrated that vitamin D deficiency was more common in patients with Ff and ff genotypes and less common in patients with FF genotype.

The extent of collateralization was the highest in FF, intermediate in Ff genotype, and the lowest in ff, suggesting that* FokI* may be functional polymorphism of VDR in collateralization. Few studies have looked at what effects the* FokI* polymorphism has on VDR function. A transcription activation study performed by Arai et al. studied the activity of two proteins in transfected HeLa cells including a vitamin D response element (VDRE). Their results showed that the shorter VDR protein was the more active form compared to the long variant; an increase of approximately 1.7-fold [48]. Using a reporter gene assay, Jurutka et al. also demonstrated that a natural polymorphic variant of human VDR, resulting from missing a* FokI* restriction site, which lacks the first three amino acids, interacted more efficiently with the human basal transcription factor IIB (TFIIB). They further suggested that the F-variant is more effective than f-variant as a transcription factor [49].

The cross-sectional design of our study limited our ability to determine the role* FokI* polymorphism may play in vascular collateralization. Despite the limitation of our study, angiographic and laboratory data are unique to our study allowing us to control most confounders. Another strength of our study is the homogeneity of population that allowed us to effectively evaluate vitamin D and the VDR gene variant in relation to cardiovascular risk factors, severity of CAD, and collateralization.

## 5. Conclusion

From our study we suggest that* FokI* polymorphism may predispose patients to CAD via vitamin D deficiency. Maintaining a normal vitamin D status should be a high priority in general population. Likewise, vitamin D supplementation may be beneficial in the prevention of CAD in patients with an increased risk for CAD. The* FokI* VDR polymorphism may have an independent association with collateralization, suggesting that genetic predisposition may contribute to different degrees of collateralization, independent of vitamin D deficiency.

Identification of the genetic basis and the pathways involved in CAD can herald a new era in diagnosis, treatment, and prevention of this disease. It is well established that functional coronary collateral arteries are of paramount importance in the prognosis of CAD. Our study may unveil a new aspect of the* FokI* polymorphism and the role of the VDR/vitamin D complex in collateral development.

## Figures and Tables

**Table 1:** Demographic and laboratory characteristics of the patients with different severity of coronary artery disease.

	Severity of CAD^¶^					
	Class 0 (*N* = 118)	Class 1 (*N* = 202)	Class 2 (*N* = 168)	Class 3 (*N* = 272)	Total (*N* = 760)	*P* value
Male % (*N*)	57.60 (68)	70.30 (142)	86.90 (146)	83.10 (226)	76.60 (582)	0.01
Age (year)*	57.46 ± 10	55.96 ± 12.48	58.42 ± 9.80	59.41 ± 9.49	57.97 ± 10.58	0.005
History of smoking % (*N*)	17.79 (21)	59.90 (121)	57.14 (96)	62.86 (171)	53.81 (409)	0.001
BMI > 30 kg/m^2^	18.64 (22)	22.70 (46)	26.19 (44)	26.10 (71)	24.07 (183)	0.01
HTN (140/90)	42.37 (50)	54.45 (110)	48.80 (82)	52.94 (144)	50.78 (386)	0.17
FBS > 110 mg/dL	6.80 (8)	13.90 (28)	13.10 (22)	15.10 (41)	13.0 (99)	0.15
TG > 150 mg/dL	14.40 (17)	33.66 (68)	34.52 (58)	42.27 (115)	33.94 (258)	0.01
LDL > 160 mg/dL	3.38 (4)	7.42 (15)	6.54 (11)	8.82 (24)	7.10 (54)	0.2
HDL < 35 mg/dL	3.38 (4)	20.29 (41)	27.38 (46)	28.67 (78)	22.23 (169)	0.001
Total cholesterol > 200 mg/dL	18.64 (22)	22.77 (46)	26.19 (44)	26.83 (73)	24.34 (185)	0.29
Severe vitamin D deficiency < 10 ng/mL	56.77 (67)	61.88 (125)	74.40 (125)	78.30 (213)	69.73 (530)	0.001
Vitamin D levels (ng/mL)*	8.17 ± 5.06	8.03 ± 5.35	7.68 ± 5.83	5.77 ± 5.24	7.19 ± 5.24	0.001
FF genotype	64.4 (76)	51.5 (104)	48.8 (82)	55.9 (152)	54.47 (414)	0.05
ff genotype	6.8 (8)	7.9 (16)	7.1 (12)	8.1 (22)	7.63 (58)	0.9
Ff genotype	28.8 (34)	40.6 (82)	44.0 (74)	36.0 (98)	37.89 (288)	0.04
Left ventricular ejection fraction*	49.45 ± 8.49	49.47 ± 10.23	49.35 ± 9.05	45.08 ± 9.26	47.77 ± 9.59	0.001

*Mean ± standard deviation.

^¶^CAD: coronary artery disease, BMI: body mass index, FBS: fasting blood sugar, *FokI* VDR genotypes: FF, ff, Ff, HTN: hypertension, HDL: high-density lipoprotein, LDL: low-density lipoprotein, and TG: triglyceride.

**Table 2:** Baseline characteristics of patients by VDR genotype.

Characteristics	The FOKI VDR genotype, % (*N*)			*P* value
	FF	ff	Ff	
	54.5 (414)	7.6 (58)	37.89 (288)	
Age (year)*	57.30 ± 10.52	55.14 ± 9.43	59.48 ± 10.69	0.003
Smoking	52.89 (219)	55.17 (32)	54.86 (158)	0.3
HTN (140/90)	50.48 (209)	53.44 (31)	50.69 (146)	0.3
BMI (kg/m^2^) > 30	21.01 (87)	34.48 (20)	26.38 (76)	0.01
FBS (mg/dL) > 110	9.66 (40)	34.48 (20)	13.54 (39)	0.01
TG (mg/dL) > 150	31.64 (131)	56.89 (33)	32.63 (94)	0.02
LDL (mg/dL) > 160	6.76 (28)	8.62 (5)	7.29 (21)	0.5
HDL (mg/dL) < 35	21.73 (90)	22.41 (13)	22.91 (66)	0.6
Total cholesterol (mg/dL) > 200	12.56 (52)	53.44 (31)	35.41 (102)	0.01
Ejection fraction (%)*	47.80 ± 10.72	42.55 ± 8.07	47.78 ± 8.48	0.03

*Mean ± standard deviation.

BMI: body mass index, FBS: fasting blood sugar, HTN: hypertension, TG: triglyceride, HDL: high density lipoprotein cholesterol, and LDL: low density lipoprotein.

**Table 3:** Vitamin D status and CAD class by VDR genotype.

Characteristics	The FOKI VDR genotype, % (*N*)			*P* value
	FF	ff	Ff	
	54.5 (414)	7.6 (58)	37.9 (288)	
Coronary artery disease				
Class 0	18.35 (76)	13.80 (8)	11.80 (34)	0.8
Class 1	25.10 (104)	27.60 (16)	28.50 (82)	0.8
Class 2	19.80 (82)	20.70 (12)	25.50 (74)	0.8
Class 3	36.70 (152)	37.90 (22)	34.00 (98)	0.7
Severe vitamin D deficiency	56.76 (235)	84.48 (49)	85.41 (246)	0.01
Well collateralization	80.19 (332)	51.72 (30)	63.88 (184)	0.01

*Mean ± standard deviation.

Patients were grouped to four classes based on luminal stenosis and the number of coronary arteries involved. In class 0, luminal stenosis was less than 50% and considered normal. In all other classes, whereluminal stenosis was more than 50%, the participants were considered CAD patients: in class 1, luminal stenosis was more than 50% in one artery; in class 2, luminal stenosis was more than 50% in two arteries; in class 3, luminalstenosis was more than 50% in three arteries.

Well collateralization: based on Rentrop scores, patients were categorized into either a poor-collateralization group (grades 0-1) or a well-collateralization group (grades 2-3).

Severe vitamin D deficiency: serum 25(OH)D_3_ level less than 10 ng/mL was considered as severe vitamin D deficiency.
